# Oligodendrocyte-specific *fus* depletion preserves CA1 single-unit fidelity and stabilizes network dynamics during chronic recording

**DOI:** 10.1088/1741-2552/ae4d8b

**Published:** 2026-03-13

**Authors:** Steven M Wellman, Kelly Guzman, Naofumi Suematsu, Teresa Thai, Te-Hsuan Tung, Camila Garcia Padilla, Sadhana Sridhar, Keying Chen, Franca Cambi, Takashi D Y Kozai

**Affiliations:** 1 Department of Bioengineering, University of Pittsburgh, Pittsburgh, PA 15213, United States of America; 2 Center for Neural Basis of Cognition, Pittsburgh, PA 15213, United States of America; 3 Veterans Administration Pittsburgh, Pittsburgh, PA 15213, United States of America; 4 Department of Neurology, University of Pittsburgh, Pittsburgh, PA 15213, United States of America; 5 Center for Neuroscience, University of Pittsburgh, Pittsburgh, PA 15213, United States of America; 6 McGowan Institute of Regenerative Medicine, University of Pittsburgh, Pittsburgh, PA 15213, United States of America; 7 Neuroscience Institute, Carnegie Mellon University, Pittsburgh, PA, United States of America; 8 Department of Molecular Cell Physiology, Kyoto Prefectural University of Medicine, Kyoto 602-8566, Japan

**Keywords:** interlaminar connectivity, functional connectivity, brain–computer interface, neural interface, biocompatibility, neural recording

## Abstract

**Objective.:**

Loss of oligodendrocytes (OLs) and myelin impairs neuronal firing and network stability, whereas enhancing oligodendrogenesis with clemastine improves electrophysiological stability in cortex and, to a lesser extent, hippocampus. Conditional depletion of *Fus* in OLs (*Fus*^OL^cKO) drives developmentally regulated increases in myelin thickness via enhanced cholesterol biosynthesis. Here, we investigated whether *Fus*-depleted OLs differentially affected long-term extracellular recordings across cortical layers and hippocampal CA1.

**Approach.:**

We performed chronic electrophysiological recordings in visual cortex and CA1 of *Fus*^OL^cKO mice and littermate controls over 16 weeks, combined with endpoint histology.

**Main results.:**

In *Fus*^OL^cKO mice, visually-evoked single-unit detectability and firing rate in CA1 increased relative to wild-type littermates, whereas cortical recordings showed no improvement. At the population level, *Fus*^OL^cKO cortex exhibited reduced firing rates and lower functional connectivity, indicating altered network dynamics. Post-mortem analysis revealed higher neuron density in recorded cortical regions acutely and greater excitatory synapse density in CA1 of *Fus*^OL^cKO mice without significant changes in myelin profiles.

**Significance.:**

*Fus* depletion in OLs enhances chronic hippocampal recordings but disrupts cortical network communication. These region-dependent effects highlight a differential role of OLs in supporting single-cell reliability versus population-level dynamics, offering novel insights into the interplay between oligodendrocytes, neural networks, and recording stability.

## 1. Introduction

Oligodendrocytes (OLs) contribute to neural circuit function by facilitating saltatory conduction and providing metabolic and neurotrophic support to axons [1–6]. Specifically, myelin sheaths produced by OLs support the rapid transmission of neuronal activity by electrically insulating axons [3, 7]. These multilamellar membranes, enriched in cholesterol and other lipids, reduce ion leakage, conserve neuronal energy and lower the energetic cost of repolarization, while directly supplying metabolic substrates such as lactate [1, 8, 9]. Given their role in maintaining axonal integrity, OLs and myelin are highly vulnerable to traumatic brain injury (TBI) and neuroinflammation, which can induce demyelination, OL degeneration, and impaired maturation of OL precursor cells [10–13]. Such disruptions compromise axonal health and neurodegeneration [14, 15], network dysfunction [12, 16], and lead to behavioral deficits [17]. Preserving or enhancing OL and myelin function may be essential for preventing axonal degeneration, maintaining network integrity, and promoting functional recovery in neurodegenerative conditions [7, 18, 19].

Disruption of OL function or demyelination in response to injury or inflammation negatively impacts neuronal activity and cortical signal propagation [20–22]. For example, cuprizone-induced OL depletion and demyelination degrades single-unit (SU) detectability, alters oscillatory patterns, and disrupts laminar signaling in the cortex [12]. Conversely, enhancing oligodendrocyte function, such as through pharmacological treatment of clemastine, significantly improves electrophysiological stability by promoting neuronal survival and increasing cortical connectivity [23]. These findings support a critical role for OLs and myelin in sustaining long-term neuronal activity and circuit-level function, particularly in disease contexts [19].

Despite the well-established roles of OLs and myelin in regulating neuronal activity as well as their susceptibility to neuroinflammation and TBI, the heterogeneity of oligodendrocytes across brain regions and the distinct molecular pathways by which they support neuronal electrophysiology remains poorly understood [18, 24, 25]. Mice with conditional depletion of the fused in sarcoma (*Fus)* gene within oligodendrocytes, termed *Fus*^OL^cKO, exhibit increased myelin thickness driven by enhanced cholesterol biosynthesis in white matter tracts without changes in OL numbers [26]. Selectively manipulating the *Fus* gene in this way would enable better understanding of the molecular mechanisms underlying myelin regulation and their impact on electrophysiological properties of neurons across different brain regions. This model would be suitable for examining the effects of myelin function in isolation, without confounding effects from changes in OL density and new oligodendrogenesis, such as those induced by clemastine. Furthermore, OLs along with astrocytes represent a source of extracellular cholesterol for adult neurons, hence, the higher cholesterol content in the *Fus*^OL^cKO may play an important neuroprotective role [27, 28]. Additionally, different brain anatomical and functional regions show an enrichment of specific subclusters of OLs [29] and restricted OL’s cholesterol and lipid synthesis [30]. Mice with conditional depletion of *Fus* [26] represent a model of a functional link between hypermyelination, cholesterol content, and neuronal activity.

In this study, we used the *Fus*^OL^cKO mouse model to investigate how oligodendrocyte-specific *Fus* deletion affects sensory-evoked neuronal electrophysiology and functional connectivity within cortical and hippocampal circuits, regions which are enriched with different subclusters of mature OLs and exhibit different myelin physiology in relation to activity-dependent myelination [31]. Given that pharmacological enhancement of myelination via clemastine preferentially improves cortical, but not CA1, electrophysiology [23], we recorded neuronal activity from both the visual V1 cortex and the underlying CA1 to assess region-specific effects of *Fus* deletion [23]. Electrophysiological recordings were acquired from *Fus*^OL^cKO mice and their wild-type (WT) littermate controls over a 16 week recording period. *Fus*^OL^cKO mice enhanced the detection, signal strength, and firing rates of individually recorded neurons within the CA1 region of the hippocampus, but not cortex. *Fus*^OL^cKO mice also exhibited reduced population-level firing rates in CA1, accompanied by decreased functional connectivity within the cortex, indicating a disruption in network-wide communication. Post-mortem immunohistochemistry revealed enhanced preservation of neurons within the recorded cortical area of *Fus*^OL^cKO mice compared to WT controls, suggesting that neuroprotection afforded by the oligodendrocyte-specific deletion of *Fus*, contributed to improved chronic electrophysiological recording outcomes.

## 2. Methods

The use of animals in this study followed the guidelines of the Institutional Animal Care and Use Committees (IACUC) of the Veterans Administration Pittsburgh Health Sciences (Animal Welfare Assurance number A3376–01) and of the University of Pittsburgh (Animal Welfare Assurance number A3187–01) and was approved by IACUC under protocols #0321 and #15043715. All mice were bred and housed in a barrier facility with a 12 h light/dark cycle. Food and water were available ad libitum. The *Fus*^OL^cKO line used for these studies was previously published [26] and was maintained by breeding *Fus*^fl/fl^/Cnp^cre/+^ (referred to as *Fus*^OL^cKO) with the *Fus*^fl/fl^ littermates (referred to as WT) for the experiments. Genotyping to detect *Fus*^fl/fl^ and Cnp^cre^ was performed as described [26]. Both males and females were used for all the analyses.

### 2.1. Surgical electrode implantation

Single-shank Michigan-style electrodes (A16–3 mm-100–703-CM16LP) were inserted into the left primary monocular visual cortex (V1) of 2 month-old *Fus*^OL^cKO mice (*n* = 6) and WT (*n* = 6). The probes extended from the pial surface through the V1 to the CA1 region of the hippocampus and recorded both spontaneous and evoked neural activity. The position in the CA1 subfield of the hippocampus was experimentally validated by visualization of the tract after acute implantation in *n* = 2 mice. Procedures for surgical electrode implantation were performed as described previously [32]. Briefly, an anesthetic cocktail of xylazine (7 mg kg^−1^) and ketamine (75 mg kg^−1^) were injected intraperitoneally (I.P.) for surgery sedation prior to fixing the animal onto a stereotaxic frame. Hair, skin, and connective tissue were removed from the top of the skull to reveal the site of surgical implantation. Vetbond adhesive was used to dry the surface of the skull prior to drilling and provide a supportive grip for a dental cement head cap. Three bone screw holes were drilled (two over both motor cortices and one over the contralateral visual cortex) prior to insertion of 4 mm long, 0.86 mm diameter stainless steel bone screws (Fine Science Tools, British Columbia, Canada) for wrapping of ground and reference wires. The ground wire was wrapped around the bone screw over the ipsilateral motor cortex while the reference wire was wrapped over the bone screws located above the contralateral motor and visual cortex. A drill-sized craniotomy positioned at 1 mm anterior to Lambda and 1.5 mm lateral to midline was made using a high-speed dental drill and 0.7 mm drill bit. Saline was periodically applied to prevent thermal damage due to drilling. A microelectrode was perpendicularly inserted at a speed of 15 mm s^−1^ using a DC motor-controller (C-863, Physik Instrumente, Karlsruhe, Germany). To record from the hippocampus, the electrode was inserted to a cortical depth of 1600 *μ*m, visually confirming that the last contact site disappeared beneath the pial surface. A silicone elastomer (Kwik-Sil) was used to fill the craniotomy around the electrode prior to sealing with a dental cement head cap. Body temperature was maintained and monitored using a rectangular heating pad (Deltaphase isothermal pad, Braintree Scientific Inc., Braintree, MA). Mice were given an I.P. injection of ketofen (5 mg kg^−1^) on the day of surgery and up to two days after for post-operative recovery.

### 2.2. Electrophysiological recordings

Electrophysiological recordings were conducted inside a grounded Faraday cage to prevent electrical interference from environmental noise as described previously [33–37]. Mice were situated on a rotating platform for awake, head-fixed recording. Trials to obtain spontaneous neural activity were recorded in a dark room. Visually-evoked neural activity was stimulated using the MATLAB-based Psychophysics toolbox on a 24″ LCD (V243H, Acer. Xizhi, New Taipei City, Taiwan) monitor located 20 cm from the contralateral eye of the mouse covering a 60° wide by 60° high visual field. A drifting gradient of solid white and black bars were presented and synchronized with the neural recording system (RX7, Tucker-Davis Technologies, Alachua, FL) at a sample rate of 24 414 Hz. Each white and black grating was presented for 1 s (rotated in 135° increments), separated by 1 s of a dark screen, and repeated for a total of 64 trials per recording session. Total recording was over 16 weeks, with daily activity measured for the first 14 d, followed by weekly recording for the remaining 14 weeks.

### 2.3. Electrophysiological data processing

#### 2.3.1. Current source density (CSD)

CSD was used to identify layer 4 along the length of the electrode following evoked activity within the visual cortex. CSD plots were generated by computing the average evoked (stimulus-locked) local field potential (LFP) for each electrode site, smoothing the signal across all electrode sites, and then calculating the second spatial derivative. CSDs were averaged across 64 stimulus trials and layer 4 was identified as an inversion in LFP polarity within the first 100 ms following stimulus onset. Cortical drift and the magnitude change in drift of implanted arrays over time was reported as the average change in layer 4 depth relative to depth at day 0 (day of surgery). All electrophysiological evaluation between different cortical depths occurred following alignment of all animals in each group for each day to their corresponding layer 4 depth. Hippocampal recording was identified by the depth and the presence of activity below the electrically silent white matter tracts.

#### 2.3.2. SU sorting and analysis

Processing of raw signal data occurred offline using a custom MATLAB script modified from previously published methods [33]. Data was passed through a Butterworth filter with a passband from 2 to 300 Hz to produce data containing information on LFPs and 0.3–5 kHz to isolate spiking information. Common average referencing was applied to the data as previously described [38]. A fixed threshold value of 3.5 standard deviations below the mean was used to identify potential neuronal SU and multi-unit (MU) activity. Only channels which exhibited a signal-to-noise ratio (SNR) >2 were considered for SU sorting. SNR were calculated by dividing the peak-to-peak amplitude of each single unit by the noise and reported as average SNR and average SNR per active site (electrode channels reporting detection of SU) over time. Sortable single units were confirmed by observing the quality and shape of neuronal waveforms, auto-correlograms, and peri-stimulus time histograms (PSTHs) with 50 ms bins. SU yield was calculated as the percentage of electrode sites (out of 16) with at least one identifiable single unit. The noise floor for each electrode site was taken as two times the standard deviation (2 × STD) of the entire recorded data stream after removing all threshold crossing events. Finally, the SNR of any channels without a sortable SU waveform were reported as zero for the purposes of calculating averages.

Putative excitatory and inhibitory units were classified based on the trough-to-peak interval (TPI) as previously described [39]. According to our whole dataset with the Gaussian mixture model fitting, we decided to use a threshold of 0.51 ms to separate units into excitatory (TPI > 0.51 ms, *n* = 2934) and inhibitory cells (TPI < 0.51 ms, *n* = 853). After the excitatory and inhibitory units were classified, unit yield (ratio of recording sites identifying excitatory/inhibitory units in total channel counts) was calculated for each animal and each day. Signal-to-noise firing rate ratio (SNFRR) for each unit was calculated as described in previous publications [40]:

(1) $$SNFRR=\frac{\mu_{ON}-\mu_{OFF}}{\frac{1}{2}\left(\sigma_{ON}+\sigma_{OFF}\right)}$$

where $\mu_{ON}$ and $\mu_{OFF}$ indicate average firing rate and standard deviation (across 64 trials) during ON (stimulation) and OFF (pre-stimulation) periods. SNFRR is a normalized firing rate change relative to each spontaneous firing rate, and its positive value corresponds to an increase in firing rate by visual stimulation. To evaluate the modulation magnitude of neural activity by visual stimulation, |SNFRR| was used.

#### 2.3.3. MU analysis

MU activity was measured as any threshold crossing event that occurred within the 1 s period after each stimulus-locked trigger or pseudotrigger was recorded. PSTHs of 50 ms bin size were generated to gauge the dynamics of MU activity. MU firing rate was measured as the average number of threshold crossing events within a 1 s period after each stimulus presentation or pseudotrigger. Evoked MU activity was evaluated by calculating MU yield and SNFRR. Parameters for MU spike counts involved varying the temporal bin size and latency after stimulus presentation from 0 to 1 s in length via 1 ms increments in order to evaluate MU yield and SNFRR. MU yield was defined as the number of electrode sites that had a significantly different spike count (p < 0.05 determined by a paired *t*-test) for a given bin size and latency following stimulus presentation (stim ON) compared to spike counts within that same bin size immediately before stimulation (stim OFF). SNFRR measured the difference in the firing rate of MU activity before and after stimulus relative to the average standard deviation between each stimulus condition (equation (1)).

#### 2.3.4. Laminar coherence analysis

Electrophysiological activity within and between different laminar depths was evaluated by calculating the magnitude-squared coherence, described previously [41]. Coherence is a quantitative description of the similarity between two signals based on their frequency-dependent responses. Coherence was reported as a value between 0 and 1, with 0 indicating no relationship and 1 indicating a perfect linear relationship between two corresponding signals. Coherence was calculated as follows:

(2) $$C_{xy}(f)=\frac{S_{xy}(f)}{\sqrt{S_{xx}(f)S_{yy}(f)}}$$

(3) $$\Delta C_{xy}(f)=C_{xy}^{E}(f)-C_{xy}^{RS}(f)$$

where $C_{xy}(f)$ is the coherence, $S_{xy}(f)$ is the cross spectrum, and $S_{xx}(f)$ and $S_{yy}(f)$ are the autospectra of LFP activity between electrode sites x and y, respectively. Normalized coherence $\Delta C_{xy}(f)$ was calculated by taking the difference between evoked coherence $C_{xy}^{E}(f)$ and resting-state coherence $C_{xy}^{RS}(f)$. Coherence calculations were performed within 1 s intervals following each stimulus presentation or pseudotrigger, at a half-bandwidth of 3 Hz and a taper number of 5, and then averaged across all trials. Coherence between different cortical regions was assessed by comparing the average coherence of all electrode sites located within each respective layer. Coherence values for a specific frequency band were reported as the average coherence within that frequency range across all animals within each group.

#### 2.3.5. Phase-amplitude coupling (PAC) analysis

To quantify the directionality of neuronal circuit connections, PAC was calculated based on Kullback–Leibler (KL) formula as previously described [42, 43]. The raw signal was processed with a band-pass filtered for specific LFP frequencies f. The Hilbert transform was applied to output the time series of the phase component from the slow frequency LFP activity in Channel X, denoted as $\phi_{X}\left(t,f_{X}\right)$ where t represents time and $f_{X}$ represents phase frequencies 2,3,4…20Hz. The time series of the amplitude component at high frequency LFP activity in channel Y $\left(A_{Y}\left(t,f_{Y}\right)\right)$ was extracted using Morlet wavelet transform (5 cycles) per frequency, using amplitude frequencies $f_{Y}=30,31,32\ldots 90\;Hz$.

Next, we calculated the amplitude of high frequency LFP oscillation in Channel Y at each given slow frequency LFP activity in Channel X, resulting in the composite time series $\phi_{X}\left(t,f_{X}\right),\;A_{Y}\left(t,f_{Y}\right)$. The phases were binned every 18° (*N* = total number of phase bins, 20) and the amplitude at each phase bin (i) was averaged to give $A_{Y}{\left(t,f_{Y}\right)}_{\phi_{X}\left(t,f_{X}\right)}(i)$. Lastly, normalizing by the sum of all bins, resulted in the normalized amplitude distribution over phases $P\left(i,f_{X},f_{Y}\right)$,

(4) $$P\left(i,f_{X},f_{Y}\right)=\frac{A_{Y}{\left(t,f_{Y}\right)}_{\phi_{X}\left(t,f_{X}\right)}(i)}{\sum_{k=1}^{N}\;A_{Y}{\left(t,f_{Y}\right)}_{\phi_{X}\left(t,f_{X}\right)}(k)}.$$

If there is no PAC between channels X and Y, the normalized amplitude distribution $P\left(i,f_{X},f_{Y}\right)$ would result in a uniform distribution. Further, the joint entropy defined as $H\left(f_{X},f_{Y}\right)$ measures the amount of deviation of the uniform distribution given by the following:

(5) $$H\left(f_{X},f_{Y}\right)=-\sum_{i=1}^{N}P\left(i,f_{X},f_{Y}\right)\;log\left(P\left(i,f_{X},f_{Y}\right)\right).$$

At the maximum joint entropy $H_{o}=logN^{2}$, the normalized amplitude distribution $P\left(i,f_{X},f_{Y}\right)$ is uniform. We then calculated the difference between $H\left(f_{X},f_{Y}\right)$ and $H_{o}$ using the KL distance formula. The modulation index (MI) of PAC was then calculated by dividing the KL distance by the uniform distribution $H_{o}$.

(6) $$MI\left(f_{X},f_{Y}\right)=\frac{H\left(f_{X},f_{Y}\right)-H_{o}}{H_{o}}.$$

When Channel X=ChannelY, a higher MI value of PAC indicates stronger LFP synchronization coupling between the low frequency phase and high frequency amplitude and increased activation level of the intralaminar network. When Channel X≠ChannelY, a larger MI value of PAC suggests enhanced LFP synchronization coupling between the low frequency phase in the upstream layer and the high frequency amplitude in the downstream layer, resulting in an increase in the directional interlaminar circuitry.

### 2.4. Electrochemical impedance spectroscopy

Electrochemical impedance was measured prior to each recording session. Mice were awake and head-fixed on a rotating platform while the implanted electrode was connected to an Autolab potentiostat along with a 16-channel multiplexer. Impedances were measured from each channel using a 10 mV RMS sine wave ranging from 10 Hz to 32 kHz. Impedances were reported as the average impedance at 1 kHz over all animals for each day unless otherwise noted.

### 2.5. Immunohistochemistry

Mouse brains were intracardially perfused with 4% paraformaldehyde (PFA), post-fixed in 4% PFA for 18 h, and kept in 30% sucrose at 4 °C until samples achieved equilibration by sinking to the bottom of the solution. Brains were frozen while embedding with optimum cutting temperature media directly on the headpiece of the cryostat (Leica) and sectioned horizontally at 14 *μ*m thickness starting from the convexity of the brain down to 800 *μ*m below the cortex. To evaluate the CA1 subregions, 14 *μ*m sections were collected between 900 *μ*m and 1600 *μ*m. To help identify the probe tract, *n* = 2 per genotype were sectioned between 2000 and 2300 *μ*m. Sections adhered to positively charged slides were permeabilized and blocked using 0.1% Triton-X with 10% normal goat serum in PBS at room temperature for 1 h and incubated with primary antibodies to CC1 (1:100, Millipore, Burlington, Massachusetts USA, #OP80), Olig2 (1:200 Millipore, Burlington, Massachusetts USA, #MABN50), NG2 (1:100, Millipore, Burlington, Massachusetts USA, #AB5320), GFAP (1:1000 Abcam, Cambridge, Massachusetts USA, #AB4674), Iba-1 (1:1000 Wako, Richmond, Virginia USA, #019–19741), MBP (1:500 Abcam, Cambridge, Massachusetts USA, #AB40390), NeuN (1:200 Millipore, Burlington, Massachusetts USA, #MAB377), Neurofilament (NF-200) (1:200, Sigma-Aldrich, St. Louis, Missouri USA #N4142), vGlut1 (1:200 Sigma Aldrich, St. Louis Missouri, USA, #AB5905) and PSD95 (1:300, Abcam, Cambridge, Massachusetts, USA, #AB18258) overnight at 4 °C. Alexa Fluor 488/594 conjugated secondary Ab (1:300 Jackson ImmunoResearch, West Grove, Pennsylvania USA # 115–585-003, # 111–605-003, #111–545-003), IgG2b isotope specific (1:300 anti-mouse 488 Jackson ImmunoResearch, West Grove, Pennsylvania USA #115–545-207) and 633 anti-chicken (1:300 Thermo Fisher Scientific, Waltham, Massachusetts #a21103) were reacted for 1 h at room temperature. Sections were washed and allowed to dry overnight in the dark before mounting with Fluoromount-G and DAPI (SouthernBiotech, Birmingham, Alabama USA, #0100–20). For synaptic staining, to keep the refractive index uniform through the section and surrounding materials we used Pro Long Glass as mounting agent which has a refractive index of 1.552 after 48 h of curing (ThermoFisher Scientific, Waltham, Massachusetts, USA, #P36982) and 1.5H coverslips (Thor Labs CG15KH1).

### 2.6. Imaging and data analysis

For cellular markers, 20x TIFF images were captured over the probe site and at an equivalent area in the contralateral hemisphere or baseline tissue with a Nikon TiE epifluorescent microscope (Melville, NY USA) with NIS Element software. For the horizontal sections, gray scale, individual layer images were cropped to square and loaded into MATLAB using a previously published I.N.T.E.N.S.I.T.Y. MATLAB script where the binning was applied around the probe site [44, 45]. Three bins were applied 0–50 *μ*m, 50–100 *μ*m and 100–150 *μ*m extending from the center of the probe site or image of non-implanted tissue. Labeled cells were counted per bin at 400–800 *μ*m through the cortical depth per brain. For intensity analysis, images were acquired by collecting 20x z-stack fields with 9 steps (1 *μ*m step size) using Nikon A1R Confocal microscope (Melville, NY, USA) with NIS Elements software. The sum intensity projections (SUMIP) were processed through MATLAB by applying 10 *μ*m bins up to 15 bins concentrically around the site of probe insertion, contralateral tissue, or baseline tissue in non-implanted mice. Data were expressed as the fold intensity change at the probe site over the intensity in the corresponding area in the contralateral hemisphere [44].

Excitatory synapses were quantified by counting appositions of pre-synaptic puncta stained with vGlut1 and post-synaptic puncta stained with PSD95 that are <200 nm apart in the CA1 [46]. This analysis was performed on SIM (Structure Illumination Microscopy, Nikon A1R) super-resolution images [47] using a workflow in the Nikon General Analysis (GA3) software, which was developed in collaboration with Nikon software specialist.

### 2.7. Statistics

Changes in recording metrics between *Fus*^OL^cKO and WT mice were analyzed using two-way repeated-measures ANOVA (rmANOVA). Factors included genotype (*Fus*^OL^cKO vs WT) and time (days post-implantation), with group × time interactions evaluated for significance. Repeated measurements within animals were accounted for through the repeated-measures design. Post-hoc comparisons between genotypes at individual timepoints were performed where appropriate and summarized using effect size estimates (Cohen’s d), with corresponding *p* values corrected for multiple testing using a Bonferroni-style adjustment. For histological analyses, one-way ANOVA or *t*-tests (p < 0.05) were used to compare tissue staining between *Fus*^OL^cKO and WT mice. When multiple pairwise comparisons were required, post-hoc *t*-tests with Bonferroni correction were applied to control for type I errors.

## 3. Results

To assess how OL-specific deletion of the *Fus* gene affects neuronal electrophysiology and local circuit connectivity, extracellular recordings were acquired from intracortical microelectrodes implanted in *Fus*^OL^cKO mice (figure 1(A)). A 16-channel Michigan-style linear electrode array was implanted to a depth of 1.6 mm, targeting both the primary visual cortex (V1) and the CA1 subregion of the hippocampus. WT littermates were similarly implanted and served as controls. Recordings were collected weekly over 16 weeks, and immunohistochemistry at 2- and 16-weeks post-implantation contextualized the data within the electrode-tissue environment. CSD in response to sensory stimulation tracked the cortical layer 4 sink over time (figure 1(B)). Minimal electrode drift (~100 *μ*m after the first week) allowed consistent analysis of intra- and interlaminar activity throughout the study.

Although both *Fus*^OL^cKO and WT mice showed the expected initial decline in electrophysiological performance, differences emerged over time. The overall percentage of electrode channels detecting discrete neuronal waveforms (recording yield) differed significantly across time and by genotype, with *Fus*^OL^cKO mice exhibiting higher recording-site yield compared to WT mice (figure 1(C), time: *F* = 31.9, *p* = 3.77 × 10^−8^; genotype: *F* = 5.45, *p* = 0.02; interaction: *F* = 2.9, *p* = 0.09, two-way rmANOVA). The SNR across all channels varied significantly over time but did not differ by genotype, and no interaction of genotype and time was observed (figure 1(D), time: *F* = 7.49, *p* = 0.0066; genotype: *F* = 1.08, *p* = 0.3; interaction: *F* = 0.07, *p* = 0.786, two-way rmANOVA). To further understand how OL-specific *Fus* deletion affects recording quality at the most reliable sites, we examined ‘active sites’, defined as channels detecting at least one discrete neuronal waveform per recording day. SNR at active sites differed significantly by genotype, with *Fus*^OL^cKO mice exhibiting higher SNR per active site overall (figure 1(e), *F* = 7.65, *p* = 0.006, two-way rmANOVA). SNR at active sites also differed significantly over time, and a significant interaction effect indicated that the temporal evolution of SNR differed between genotypes (time: *F* = 8.84, *p* = 0.003; interaction: *F* = 5.96, *p* = 0.015). Signal amplitude did not differ significantly by genotype or time and showed no significant interaction effect (figure 1(f), genotype: *F* = 1.02, *p* = 0.312; time: *F* = 3.48, *p* = 0.063; interaction: *F* = 2.18, *p* = 0.141, two-way rmANOVA). Noise floor amplitude was also similar across time and genotype (figure 1(g), time: *F* = 1.73, *p* = 0.189; genotype: *F* = 2.56, *p* = 0.111; interaction: *F* = 0.19, *p* = 0.668, two-way rmANOVA). Taken together, these observations suggest that OL-specific *Fus* deletion enhances the detection and strength of recorded neuronal activity.

Electrode performance and tissue encapsulation remained comparable across genotypes. Electrochemical impedance spectroscopy showed similar impedance magnitudes across the 16 week recording period between *Fus*^OL^cKO and WT controls (figure 1(H), time: *F* = 2.28, *p* = 0.132; genotype: *F* = 2.02, *p* = 0.157; interaction: *F* = 2.70, *p* = 0.101, two-way rmANOVA), indicating comparable electrode performance and glial encapsulation. Because Johnson–Nyquist noise scales with impedance, the elevated noise floor in *Fus*^OL^cKO mice is unlikely to result from thermal noise or encapsulation. Together, these results indicate that OL-specific *Fus* deletion enhances long-term neuronal recording fidelity, likely by preserving myelin integrity and maintaining local circuit viability.

### 3.1. Fus^OL^cKO mice improve the detection of neurons within CA1 region of the hippocampus

Aberrant myelination contributes to network dys-function in many neurodegenerative disorders, but the functional consequences of increased myelin thickness following conditional *Fus* deletion in oligodendrocytes remain unclear. We hypothesized that thicker myelin in *Fus*^OL^cKO mice would enhance the detectability and strength of extracellularly recorded spikes by improving action potential propagation and axonal synchrony. To test this, we analyzed electrophysiological metrics separately in the primary visual cortex (V1) and the CA1 subregion of the hippocampus.

Recording yield in V1 varied significantly over time but did not differ by genotype (figure 2(A), time: *F* = 11.3, *p* = 0.0009; genotype: *F* = 0.81, *p* = 0.369; interaction: *F* = 3.2, *p* = 0.075, two-way rmANOVA). In contrast, CA1 yield showed a strong significant effect across time and genotype, with *Fus*^OL^cKO mice exhibiting higher recording site yield compared to WT controls (time: *F* = 22.7, *p* = 2.9 × 10^−6^; genotype: *F* = 4.39, *p* = 0.0369; interaction: *F* = 0.3, *p* = 0.584, two-way rmANOVA). SNR in V1 did not differ by genotype and showed no evidence of time-dependent genotype modulation (figure 2(B), time: *F* = 0.88, *p* = 0.35; genotype: *F* = 1.6 × 10^−7^, *p* = 1.00; interaction: *F* = 0.06, *p* = 0.807, two-way rmANOVA). In CA1, SNR varied significantly over time but did not differ by genotype, and no interaction effect was observed (time: *F* = 8.65, *p* = 0.0035; genotype: *F* = 2.00, *p* = 0.159; interaction: *F* = 0.03, *p* = 0.874, two-way rmANOVA). Together, these findings suggest that OL-specific *Fus* deletion preferentially enhances recording yield in CA1 with time-dependent fluctuations in SNR reflecting post-implantation dynamics rather than region-specific differences.

Analysis of spike amplitude and noise characteristics further supported this region-specific effect. Spike amplitudes did not differ significantly by genotype in either cortex nor CA1, and no significant interaction effects were observed in either region (figure 2(C), time in cortex: *F* = 5.47, *p* = 0.0199; genotype in cortex: *F* = 0.26, *p* = 0.612; interaction in cortex: *F* = 2.4, *p* = 0.122, time in CA1: *F* = 0.81, *p* = 0.369, genotype in CA1: *F* = 2.67, *p* = 0.103; interaction in CA1: *F* = 1.08, *p* = 0.30, two-way rmANOVA). Noise floor amplitudes similarly showed no significant genotype effects in either cortex nor CA1 (figure 2(D), time in cortex: *F* = 0.02, *p* = 0.893; genotype in cortex: *F* = 0.23, *p* = 0.636; interaction in cortex: *F* = 0.29, *p* = 0.592, time in CA1: *F* = 6.09, *p* = 0.014, genotype in CA1: *F* = 2.79, *p* = 0.096; interaction in CA1: *F* = 0.38, *p* = 0.537, two-way rmANOVA). Together, these findings indicate that conditional deletion of *Fus* in oligodendrocytes selectively enhances neuronal signal detection in the hippocampal CA1 region, with only minor or transient effects observed in cortex, supporting the idea that myelin changes in *Fus*^OL^cKO mice can selectively improve axonal conduction and network excitability in specific brain regions.

### 3.2. Fus^OL^cKO mice enhance visually evoked firing rates of individual neurons within CA1 region of the hippocampus

The degree of oligodendrocyte-mediated myelination influences not only conduction velocity but also the energy efficiency and reliability of axonal transmission, particularly in long-range projections such as those targeting the hippocampus. We hypothesized that conditional *Fus* deletion in oligodendrocytes would increase neuronal firing rates relative to WT controls. Consistent with this expectation, depth-dependent differences in neuronal activity were evident in heatmaps comparing spontaneous and visually evoked firing between *Fus*^OL^cKO and WT mice (figures 3(A) and (C)). Spontaneous firing rates in cortex and CA1 varied significantly over time but did not differ significantly by genotype, with a trend toward higher spontaneous firing in CA1 of *Fus*^OL^cKO mice relative to WT controls (figure 3(B), time in cortex: *F* = 14.1, *p* = 0.0002; genotype in cortex: *F* = 1.85, *p* = 0.175; interaction in cortex: *F* = 1.40, *p* = 0.237, time in CA1: *F* = 44.7, *p* = 1.1 × 10^−10^, genotype in CA1: *F* = 3.58, *p* = 0.059; interaction in CA1: *F* = 0.36, *p* = 0.55, two-way rmANOVA). In contrast, visually evoked firing showed strong genotype-dependent modulation in CA1, but not cortex, with elevated firing rates in *Fus*^OL^cKO mice compared to WT controls (figure 3(D), time in cortex: *F* = 19.6, *p* = 1.34 × 10^−5^; genotype in cortex: *F* = 1.30, *p* = 0.254; interaction in cortex: *F* = 0.86, *p* = 0.355, time in CA1: *F* = 43.4, *p* = 1.91 × 10^−10^, genotype in CA1: *F* = 6.33, *p* = 0.012; interaction in CA1: *F* = 0.004, *p* = 0.948, two-way rmANOVA). These findings indicate that *Fus* deletion in oligodendrocytes selectively enhances visually evoked neuronal activity in the hippocampal CA1 region, supporting the idea that increased myelination improves long-range network excitability and the reliability of information transmission.

### 3.3. Fus^OL^cKO mice preserve excitatory yield and early inhibitory responsiveness in a region- and cell type-specific manner

To investigate the influence of conditional knockout of *Fus* in oligodendrocytes on neuronal subtype-specific activity, we separated putative excitatory and inhibitory units based on the TPI of their spike waveforms. A Gaussian mixture model was fit to the TPI distribution, identifying a classification threshold of 0.51 ms: units with TPI > 0.51 ms were categorized as putative excitatory, while those with TPI < 0.51 ms were categorized as putative inhibitory (figure 4(A), left). Representative waveforms for each category (mean ± 1 SD) are shown (figure 4(A), right). The overall number of putative excitatory and inhibitory units detected per animal was not significantly different between WT and *Fus*^OL^cKO mice (number of excitatory units: 127.3 ± 9.4 units in WT versus 148 ± 10.8 units in *Fus*^OL^cKO, *p* = 0.1616; number of inhibitory units: 47.4 ± 3.6 in WT versus 35.5 ± 5.6 in *Fus*^OL^cKO, *p* = 0.1161, Student’s *t*-test). However, the average proportion of inhibitory-to-excitatory units was significantly lower in *Fus*^OL^cKO mice compared to WT controls (proportion of Inh/Exc units in WT: 0.3827 ± 0.0311; proportion of Inh/Exc units in *Fus*^OL^cKO: 0.2488 ± 0.0415; *p* = 0.0252, Student’s *t*-test). Average yield of putative excitatory and inhibitory units across depth differed significantly by genotype. The main effect of genotype indicated that *Fus*^OL^cKO mice exhibited a higher yield of putative excitatory units compared to WT controls (figure 4(B), left, time: *F* = 9.23, *p* = 0.00259; genotype: *F* = 18.34, *p* = 2.49 × 10^−5^; interaction: *F* = 3.12, *p* = 0.0783; two-way rmANOVA). Conversely, WT mice displayed higher inhibitory unit yields than *Fus*^OL^cKO mice (figure 4(B), right, time: *F* = 36.7, *p* = 4.07 × 10^−9^; genotype: *F* = 9.34, *p* = 0.002 44; interaction: *F* = 2.13, *p* = 0.146; two-way rmANOVA). This suggests that *Fus* depleted oligodendrocytes may improve the detection, stability, or viability of excitatory neurons while having a more limited or suppressive effect on inhibitory unit detection.

To determine whether these differences varied across brain regions, we analyzed longitudinal unit yield in cortex and hippocampal CA1. Putative excitatory unit yield in cortex declined over time in WT mice, consistent with glial scarring or electrode drift. In contrast, *Fus*^OL^cKO mice maintained significantly higher excitatory unit over time. This was supported by a significant effect of time and genotype (figure 4(C), left, time: *F* = 12.9, *p* = 0.000 386; genotype: *F* = 14.6, *p* = 0.00016; interaction: *F* = 0.39, *p* = 0.533; two-way rmANOVA), indicating that excitatory yield diverged between genotypes over time. For putative inhibitory units, WT mice showed higher early yield than *Fus*^OL^cKO mice. However, although the main effect of genotype and time was significant the interaction was not (figure 4(C), right, time: *F* = 32.4, *p* = 3.05 × 10^−8^; genotype: *F* = 7.94, *p* = 0.00516; interaction: *F* = 5.37, *p* = 0.0212; two-way rmANOVA) indicating that the temporal profile of inhibitory yield did not differ significantly between genotypes. Within CA1, putative excitatory unit yield was largely stable over time in both genotypes, but *Fus*^OL^cKO mice consistently exhibited higher excitatory yield than WT controls, as reflected by a significant main effect of genotype (figure 4(D), left, time: *F* = 0.0163, *p* = 0.899; genotype: *F* = 12.2, *p* = 0.000551; interaction: *F* = 0.368, *p* = 0.545; two-way rmANOVA). For putative inhibitory units in CA1, WT mice showed greater yield than *Fus*^OL^cKO mice across time, supported by a significant main effect of genotype (figure 4(D), right, time: *F* = 9.61, *p* = 0.00212; genotype: *F* = 5.39, *p* = 0.0209; interaction: *F* = 0.256, *p* = 0.613; two-way rmANOVA). These findings indicate that conditionally depleting *Fus* in oligodendrocytes preferentially stabilized excitatory signals in both cortex and hippocampus, while inhibitory units were less reliably preserved.

To evaluate functional responsiveness at the population level, we computed the absolute |SNFRR| for putative excitatory and inhibitory units across time and depth (figures 4(E) and (G)). In the cortex, putative excitatory |SNFRR| remained stable over time, as indicated by a nonsignificant effect of time (figure 4(F), left, *F* = 0.0007, *p* = 0.979, two-way rmANOVA). However, WT mice exhibited substantially greater excitatory |SNFRR| than *Fus*^OL^cKO mice across time (*F* = 19.92, *p* = 9.39 × 10^−6^), with no significant genotype × time interaction (*F* = 0.51, *p* = 0.473), indicating a consistent genotype-dependent difference in response amplitude. In hippocampal CA1, putative excitatory |SNFRR| did not differ between genotypes (figure 4(F), right, *F* = 0.35, *p* = 0.553, two-way rmANOVA) and remained stable across time (*F* = 1.81, *p* = 0.179), with no significant genotype × time interaction (*F* = 1.83, *p* = 0.177). Putative inhibitory |SNFRR| in cortex exhibited substantial fluctuations early in the recording period but showed no consistent differences between genotypes or across time (figure 4(H), left, time: *F* = 0.45, *p* = 0.503; genotype: *F* = 0.13, *p* = 0.724; interaction: *F* = 0.005, *p* = 0.945, two-way rmANOVA), indicating that inhibitory response amplitudes in cortex were similar between WT and *Fus*^OL^cKO mice and remained stable over the recording period. In contrast, inhibitory |SNFRR| in CA1 declined significantly over time in both groups (figure 4(H), right, time: *F* = 9.60, *p* = 0.0023, two-way rmANOVA). *Fus*^OL^cKO mice displayed higher inhibitory |SNFRR| early in the recording period, reflected by a significant main effect of genotype (*F* = 7.95, *p* = 0.005 36) and a significant genotype × time interaction (*F* = 6.03, *p* = 0.015). However, these early differences were not sustained chronically, and both genotypes converged toward similar long-term inhibitory response amplitudes. These data imply that early increases in inhibitory responsiveness, possibly associated with increased myelin thickness following conditional *Fus* deletion in oligodendrocytes are transient and may reflect temporary increases in conduction fidelity or excitability, while long-term benefits are preferentially conferred to excitatory neurons.

### 3.4. Fus^OL^cKO mice demonstrate reduced neuronal firing at the population level in CA1 region of hippocampus

To investigate how OL-specific *Fus* deletion alters evoked ensemble activity, we evaluated MU firing responses in *Fus*^OL^cKO mice. MU activity reflects the aggregate firing of multiple nearby neurons and provides a measure of population-level response dynamics. We characterized MU responses to visual stimulation using two metrics: MU yield and SNFRR, as previously described [12, 23, 48]. MU yield was calculated as the percentage of electrode channels exhibiting a significant change in firing rate during a period of drifting grating stimulation compared to an equivalent pre-stimulation period while SNFRR quantified the magnitude of this change for each channel. Both metrics were evaluated across a range of post-stimulus bin sizes (window durations) and latencies (time since stimulus onset) to capture dynamic responses. To visualize temporal response properties, heatmaps of average MU yield were generated across cortical and hippocampal regions (figure 5(A)). In both regions, MU responses exhibited a stereotyped onset component within 100 ms post-stimulation, followed by intermittent sustained responses up to 800 ms post-stimulation. This temporal pattern reflects both initial sensory encoding and prolonged circuit reverberations.

Next, to standardize comparisons, all between-group analyses were anchored at the bin sizes that maximized MU yield in WT mice, corresponding to 46 ms in cortex and 97 ms in CA1, consistent with prior optimization [23]. This approach ensured sensitivity to stimulus-driven responses while controlling for regional dynamics. To determine whether conditional depletion of *Fus* in oligodendrocytes altered the timing of evoked population responses, latencies were calculated as the time interval between the onset of stimulation and the first statistically measurable change in neuronal firing rate relative to spontaneous activity. Sensory response latency did not differ significantly between *Fus*^OL^cKO and WT mice in either cortex or CA1 (figure 5(B), time in cortex: *F* = 0.47, *p* = 0.492; genotype in cortex: *F* = 0.11, *p* = 0.747; interaction in cortex: *F* = 0.04, *p* = 0.844, time in CA1: *F* = 1.94, *p* = 0.165, genotype in CA1: *F* = 0.40, *p* = 0.53; interaction in CA1: *F* = 0.37, *p* = 0.542, two-way rmANOVA). We also quantified the duration of sensory-evoked MU responses by measuring the longest consecutive run of detectable activity following stimulus onset. Similar to our latency results, response duration did not differ significantly between genotypes or over time in either cortex or CA1 (*data not shown*, time in cortex: *F* = 36.6, *p* = 4.25 × 10^−9^; genotype in cortex: *F* = 0.0282, *p* = 0.867; interaction in cortex: *F* = 0.291, *p* = 0.59; time in CA1: *F* = 1.24, *p* = 0.267; genotype in CA1: *F* = 0.0384, *p* = 0.845; interaction in CA1: *F* = 0.481, *p* = 0.488, two-way rmANOVA). These results indicate that the timing and duration of sensory signal arrival and initial encoding is preserved in *Fus*^OL^cKO mice, agreeing with results obtained in previous studies [12, 23, 48]. However, clear genotype-dependent differences emerged in response magnitude. During the onset window (<100 ms from visual stimulus onset), cortical onset SNFRR varied strongly over time but did not differ significantly by genotype, nor was any significant interaction effect observed (figure 5(C), time: *F* = 87.9, *p* = 1.52 × 10^−18^; genotype: *F* = 0.76, *p* = 0.386; interaction: *F* = 3.25, *p* = 0.072, two-way rmANOVA). In contrast, onset SNFRR in CA1 exhibited a significant genotype effect, alongside strong temporal modulation and significant interaction effect (time: *F* = 43.4, *p* = 1.92 × 10^−10^; genotype: *F* = 6.16, *p* = 0.0136; interaction: *F* = 12.4; *p* = 0.000 506, two-way rmANOVA). During the sustained response window (100–800 ms post-stimulation), sustained SNFRR within the cortex varied significantly over time but did not differ by genotype (figure 5(D), time: *F* = 79.1, *p* = 4.19 × 10^−17^; genotype: *F* = 0.12, *p* = 0.731; interaction: *F* = 0.45, *p* = 0.501, two-way rmANOVA). In contrast, sustained SNFRR within CA1 exhibited strong temporal modulation, a significant main effect of genotype, and a significant interaction effect (time: *F* = 58.2, *p* = 2.94 × 10^−13^; genotype: *F* = 12.8, *p* = 0.000 394; interaction: *F* = 9.3, *p* = 0.002 49; two-way rmANOVA). These results indicate that OL-specific *Fus* deletion selectively alters both the magnitude and temporal profile of sustained sensory-evoked responses in hippocampal CA1, indicating potentially dampened network-level excitability or recurrent activity in hippocampal circuits during sensory processing.

### 3.5. Fus^OL^cKO mice demonstrate reduced laminar connectivity between cortical layers

Given that previous results showed no significant effects on single-neuron or population firing properties in cortex, we investigated whether OL-specific *Fus* deletion alters functional connectivity across cortical layers during sensory processing in *Fus*^OL^cKO mice. We used coherence analysis of LFP to quantify the similarity of neural activity between cortical depths. Functional connectivity changes during visual stimulation were expressed as ΔCoherence, comparing evoked versus spontaneous activity across frequency bands and cortical layers. Heatmaps of ΔCoherence revealed prominent differences between *Fus*^OL^cKO mice and WT at 63 d post-implantation between L2/3 and L5 within the delta (2–4 Hz) frequency band (figure 6(A)). Over a 16 week period, *Fus*^OL^cKO mice showed significantly reduced normalized coherence between these layers in the delta compared to WT, as indicated by a significant main effect of genotype (figure 6(B), *F* = 6.90, *p* = 0.0091, two-way rmANOVA). Neither the main effect of time (*F* = 0.29, *p* = 0.588) nor the genotype × time interaction (*F* = 0.001, *p* = 0.971) reached significance, indicating that the reduction in coherence was stable across time. Similarly, coherence between layer 4 and layer 2/3 within the low beta/alpha-like frequency band (8–20 Hz) was markedly reduced in *Fus*^OL^cKO mice (figure 6(C)). A significant main effect of genotype confirmed lower coherence in *Fus*^OL^cKO compared to WT mice (figure 6(D), *F* = 39.78, *p* = 1.03 × 10^−^⁹, two-way rmANOVA). Coherence also changed over time (*F* = 9.81, *p* = 0.0019), although the genotype × time interaction did not reach significance (*F* = 2.51, *p* = 0.114). These effects were most pronounced shortly after implantation and persisted at several subsequent time points. Overall, the results indicate that functional communication between key cortical layers across multiple frequency bands is reduced in *Fus*^OL^cKO mice.

These results prompted us to examine whether more subtle forms of network coordination, such as cross-frequency interactions, might also be affected by OL-specific *Fus* deletion, even in the absence of broad changes in single-neuron or population firing. We performed PAC analysis of LFPs to measure how the phase of slower oscillations modulates the amplitude of faster rhythms across cortical layers, a mechanism implicated in neural coding and network coordination. Across days of visually evoked activity, MI values for theta-gamma coupling (theta phase: 4–8 Hz; gamma amplitude: 55–70 Hz) exhibited a significant main effect of time, but no significant main effect of genotype, for coupling between L4 and L2/3 (figures 6(E) and (F), time: *F* = 15.9, *p* = 8.39 × 10^−5^; genotype: *F* = 1.15, *p* = 0.284; two-way rmANOVA). A modest genotype × time interaction was detected for L4-L2/3 coupling (*F* = 3.91, *p* = 0.0488; two-way rmANOVA), although post-hoc comparisons did not identify individual timepoints with significant between-group differences. Likewise, PAC between L2/3 and L5 showed a significant effect of time but no genotype-dependent differences (figures 6(G)–(H), time: *F* = 14.2, *p* = 1.96 × 10^−4^; genotype: *F* = 1.01, *p* = 0.317; interaction: *F* = 3.46, *p* = 0.0639, two-way rmANOVA). In contrast to the reduced interlaminar coherence observed in *Fus*^OL^cKO mice, PAC metrics remained stable across genotype, indicating that cross-frequency coupling within the cortex is largely preserved following oligodendrocyte-specific *Fus* depletion under visually evoked conditions. Overall, these findings suggest that while OL-specific *Fus* depletion affects interlaminar coherence, it does not produce widespread changes in PAC.

### 3.6. Fus^OL^cKO mice retain more cortical neurons during acute injury phase following electrode implantation

To evaluate neuronal survival and circuit integrity following acute brain perturbation, we examined cellular responses in the cortex of *Fus*^OL^cKO and WT mice via immunofluorescence at 2- and 16-weeks post-implantation. *Fus*^OL^cKO mice exhibited significantly higher densities of NeuN+ neurons within 100 *μ*m of the electrode track at 2 weeks compared to WT controls (figure 7(A), p < 0.05, one-way ANOVA), indicating enhanced neuronal survival during the acute injury phase. By 16 weeks, neuronal densities converged between genotypes, suggesting this protective effect is temporally limited to the early post-implantation period. Despite differences in neuronal survival, axonal density measured by NF200+ fluorescence intensity did not differ significantly between *Fus*^OL^cKO and WT mice at either time point (figure 7(B), p > 0.05, one-way ANOVA). Similarly, counts of CC1+ oligodendrocytes (figure 7(C), p > 0.05, one-way ANOVA) and amount of MBP+ myelin sheaths (figure 7(D), p > 0.05, one-way ANOVA) were comparable between genotypes at both 2 and 16 weeks. In order to assess glial immune response, we measured microglial and astrocytic activation near the implanted electrode. Iba-1+ fluorescence intensity, as a marker of microglial activation, did not differ significantly within 150 *μ*m of the electrode between *Fus*^OL^cKO and WT mice at either time point (figure 7(E), p > 0.05, one-way ANOVA). However, GFAP staining revealed a significant reduction in astrogliosis at 2 weeks post-implantation in *Fus*^OL^cKO mice, specifically within 30 *μ*m of the implant site, although this difference was no longer evident at 16 weeks (figure 7(F), p < 0.05, one-way ANOVA). These results indicate attenuation of the early astrocytic responses to injury without substantial effects on microglial activation. Despite using multiple approaches to localize the electrode track in the CA1 hippocampal subregion (900–1600 *μ*m), unequivocal identification was not achieved, precluding direct cellular quantification near the implant in this area. However, immunohistochemical analysis of CA1 pyramidal neurons using NeuN and MAP2 staining and myelin density with MBP in the pyramidal layer did not reveal significant genotype differences (supplementary figure 1). Collectively, immunostaining with MBP did not reveal an increase in myelin profiles. This result could reflect the low sensitivity of MBP immunostaining for subtle changes in myelin density.

Finally, cholesterol and cholesterol esters content are significantly higher in the *Fus*^OL^cKO hippocampus vs. WT at 2- and 6-months of age which spans the recording time in the present study [49]. We reasoned that cholesterol-driven hypermyelination might enhance neural function by supporting glutamatergic synaptic density in CA1 of *Fus*^OL^cKO mice (supplementary figure 2). Using postsynaptic density protein 95 (PSD95) as a marker of post-synaptic sites and vesicular glutamate transporter (vGlut1) as a presynaptic excitatory neuron marker, we applied structured illumination microscopy and NIS Element software for precise synaptic apposition quantification at 14 d post-implantation [47, 50]. *Fus*^OL^cKO mice exhibited a significant increase in excitatory synapse number compared to WT controls (p < 0.05, one-way ANOVA). These findings support a potential role of myelin and cholesterol in increasing excitatory synaptic density in CA1, which may underlie improved electrophysiological readouts by facilitating stronger excitatory transmission during early post-injury stages.

## 4. Discussion

Myelin integrity and OL-mediated metabolic support are fundamental to CA1 circuit function and hippocampal-dependent memory [1, 51, 52]. In *Fus*^OL^cKO mice, conditional deletion of *Fus* in OLs drives cholesterol-mediated hypermyelination in white matter tracts [26], allowing us to probe its effects on sensory-evoked activity and network synchrony. This study has revealed a differential impact of *Fus* depleted OLs in the cortex and hippocampus, highlighting the complexity of OL mediated support of neurons. *Fus* depleted OLs selectively enhanced single-unit (SU) detectability and firing rates in CA1 while leaving cortical SU responses unchanged, yet it reduced MU population firing in CA1, consistent with a shift toward sparser, energy-efficient coding. In the cortex, laminar coherence in delta and alpha–beta bands declined, indicating refined interlaminar synchrony. Post-mortem analysis demonstrated that better preservation of NeuN+ neurons in cortex is an early event. In the CA1 subregion, there is an increase in excitatory synapse density without changes in neuron density and myelin profiles. These results reveal that *Fus* depleted oligodendrocytes function independently from compact myelin density and can optimize individual neuron output and reshape large-scale network coordination without uniformly altering cortical excitability.

### 4.1. Regional specificity of OL Fus depletion -effect on electrophysiological enhancements

Our results reveal a striking regional specificity in how *Fus* depleted OLs alter electrophysiological properties. *Fus*^OL^cKO mice exhibit marked improvements in SU detectability within hippocampal CA1, effects that persist beyond four weeks of chronic recording (figures 2(A)–(E); figures 3(A)–(D)). By contrast, cortical SU metrics remain unchanged (figures 2(A)–(C)). These results could be attributed to the heterogeneous nature of myelin distribution in the brain, with myelin density increasing as a function of depth relative to the cortical surface [53]. Potentially, this organization is motivated by hippocampal neurons, especially in CA1, being more reliant on myelin functions for maintaining excitatory transmission and synaptic plasticity, while cortical neurons may have different compensatory mechanisms or less dependency on myelin for initiating and sustaining their activity [54]. In the current study, we did not detect a statistically significant increase in myelin density in either cortex or CA1, suggesting the possibility that other myelin mediated effects, such as specific myelin–axon interactions, especially at paranodal regions, may be playing a role [55]. This important mechanism may stabilize neural transmission and enhance signaling at the synapses in *Fus*^OL^cKO mice. Indeed, *Fus*^OL^cKO mice displayed a significantly higher density of excitatory synapses within hippocampal CA1 (supplementary figure 1). Another mechanism to account for this increase is stabilization of excitatory synapses by the higher cholesterol content associated with *Fus* depletion in the hippocampus [49] leading to higher excitability within the hippocampus [56, 57]. Lastly, genes involved in myelin wrapping, cytoskeletal organization, OL differentiation and membrane transport are upregulated in hippocampal OL [49] indicating preservation of OL identity, differentiation state, and protein transport.

Paradoxically, MU firing rates and signal-to-noise firing rate ratios in CA1 are reduced during both onset (0–100 ms) and sustained (100–800 ms) responses (figures 5(C) and (D)). This shift toward sparser MU activity, despite elevated SU performance, is consistent with an energy-efficient coding strategy supported by myelin’s reduction of ionic flux and metabolic cost [58, 59]. This paradoxical reduction in population-level firing may reflect enhanced energy efficiency. By facilitating faster, more reliable axonal conduction, myelin allows individual neurons to maintain output with fewer synchronous spikes, thereby reducing overall metabolic demand during network activity and increasing network efficiency [60]. Interestingly, the region-specific efficacy of *Fus*^OL^cKO in CA1 but not cortex contrasts with prior findings using clemastine, which improved electrophysiological signals in both regions [23]. This divergence suggests that cortical resilience may depend more on new oligodendrogenesis and new myelin segment deposition or non-myelinating functions of oligodendrocytes, such as metabolic support or neuroimmune modulation, whereas CA1 appears more sensitive to preexisting myelin content and its function in axonal connection and communications.

An additional consideration when interpreting differences in electrophysiological metrics is the well-documented influence of locomotion on neuronal activity in both the V1 cortex and CA1 hippo-campus. Locomotion can alter firing rates, reduce noise correlations, and modulate sensory and spatial encoding under head-fixed recording conditions [61–65]. *Fus*-deficient mice are known to exhibit motor hyperactivity [26]. Since motor behavior or locomotion bouts were not monitored in the present study, we cannot fully exclude the possibility that differences in behavioral state contributed to some of the physiological effects observed. Future studies incorporating locomotor tracking will be needed to isolate locomotion-dependent influences from direct consequences of OL-specific *Fus* depletion.

### 4.2. Fus depleted OLs preserve chronic SU fidelity and neuronal survival in CA1

Early neuronal preservation in the cortex and increased excitatory synapse density in hippocampal CA1 (figures 7(A) and (B); supplementary figure loco-motion 1) suggest that *Fus*^OL^cKO mice may exhibit enhanced microcircuit stability during the acute implantation period. Recording performance in the hippocampus typically suffers due to electrode penetration through heavily myelinated axon tracts projecting to CA1 pyramidal neurons. This limitation restricts studies of memory, learning, and plasticity in a region critical for cognitive function. *Fus*^OL^cKO mice show significantly improved recording performance in the hippocampus (figure 3), highlighting myelin’s role in mitigating chronic recording failure. These observations align with the concept that myelin enhances axonal efficiency by reducing sodium influx during action potentials and lowering ATP demands for repolarization [66].

*Fus* depletion in OLs selectively increases the percent of myelinated small diameter axons in the callosum [26], a population characterized by high membrane surface-to-volume ratios and correspondingly greater metabolic demand during rapid firing [59, 66]. These fine-caliber fibers are essential for precise spike timing and local circuit computation in CA1, yet are especially vulnerable to energy depletion in zones of sparse vascularization and elevated synaptic throughput [67, 68]. Microelectrodes are typically implanted from the dorsal surface, traversing axon-rich regions such as the stratum oriens and alveus, which contain myelinated Schaffer collateral projections from CA3 and extrahippocampal inputs; this trajectory disrupts and demyelinates axons critical for synaptic drive into CA1. By adding extra insulation to these metabolically sensitive axons, hypermyelination in *Fus*^OL^cKO mice reduces ionic flux and ATP requirements per action potential [66]. In parallel, oligodendrocyte-mediated transfer of glycolytic lactate and glucose via MCT1 and GLUT1 transporters provides an auxiliary substrate to buffer metabolic stress [6, 69]. This dual enhancement of structural insulation and glial metabolic support likely underpins the observed preservation of neuronal survival and SU fidelity under conditions of chronic implant stress.

Early preservation of neurons can have a positive influence on chronic recording stability. Previous studies have demonstrated strong correlations between neuronal density near recording electrodes and long-term signal quality, including improved SNR, spike amplitudes, and reduced signal degradation [70, 71]. Therefore, the transient increase in NeuN+ neuron preservation reported in *Fus*^OL^cKO mice acutely may contribute to the long-lasting improvements in SU fidelity we observed. Alternatively, because *Fus* deletion in oligodendrocytes occurs developmentally in this model, we cannot fully exclude the possibility that early-life changes in myelination or circuit maturation contribute to the acute neuronal differences we detected. Future studies employing an inducible OL-specific *Fus* knockout which allows *Fus* depletion to occur only after normal development has concluded will be critical for disentangling potential developmental effects from injury-specific mechanisms.

Despite reduced population-level firing in CA1, the preservation of excitatory unit yield indicates more stable and reliable signal detection relative to WT controls. These results demonstrate that myelin in *Fus*^OL^cKO mice may counter typical chronic recording degradation in CA1, a region vital for hippocampal-dependent cognitive processes. These findings extend prior pharmacological studies with clemastine [23] by isolating the contribution of myelin thickness itself from broader oligodendrocyte effects [26], emphasizing the importance of myelin integrity for sustaining hippocampal circuit function under chronic stress.

### 4.3. Refinement of cortical network synchrony is affected by Fus depleted OLs

In *Fus*^OL^cKO mice, cortical SU and MU activity metrics remained stable (figures 2, 3), yet interlaminar coherence was significantly reduced in delta (2–4 Hz) and alpha–beta (8–20 Hz) frequency bands (figures 6(A)–(D)). These findings indicate that *Fus* dependent myelination modulates the temporal coordination of oscillatory activity between cortical layers without broadly enhancing cortical excitability. Reduced LFP power across frequency bands in *Fus*^OL^cKO mice, particularly near the implant site, may reflect a lower need for compensatory network recruitment (figure 6). In WT mice, acute injury and local demyelination likely provoke nearby circuits to increase synchronous firing and oscillatory activity to maintain functional output, manifesting as higher LFP power. By contrast, *Fus*^OL^cKO mice exhibit preserved excitatory unit yield, early inhibitory responsiveness, and local myelin integrity, reducing the requirement for compensatory hyperactivity. Consequently, lower LFP power may indicate more efficient, balanced network function rather than diminished excitability, consistent with sustained SU fidelity and enhanced spike detection over time. These results are analogous to compensatory network recruitment observed in stroke and TBI models, where acute injury transiently elevates neuronal oscillatory power as adjacent circuits increase synchrony to maintain function [43, 72–74]. In *Fus*^OL^cKO mice, attenuated injury responses and preserved oligodendrocyte function reduce the need for such compensatory recruitment, manifesting as lower LFP power across bands over time.

Global oscillatory power across frequency bands was unchanged, consistent with prior observations that oligodendrocyte loss or pharmacological rescue primarily affect the strength of specific neuronal oscillations [12, 23]. Our results suggest that the activity of individual neurons is modulated without proportionally increasing synchrony across large neuronal populations. Hypermyelination may fine-tune local circuit dynamics while preserving the overall balance of cortical oscillations [75]. Notably, *Fus*^OL^cKO mice showed reduced laminar connectivity relative to WT controls, suggesting that single-neuron function is strengthened but coordinated communication between cortical layers may be impaired. Rather than facilitating intra- and interlaminar signaling, *Fus* depleted OLs could affect temporal misalignment across cortical circuits, potentially disrupting the integration of complex information [75]. This nuanced modulation contrasts with the preservation of firing properties at the single-neuron level, implying a selective influence on the coordination of population activity across cortical layers. Such changes in cortical network dynamics may impact cognitive functions that depend on precise interlaminar signaling, including sensory integration and higher-order processing [76, 77].

## 5. Conclusion

This study advances understanding of how OL and myelin contribute to stable SU activity and sustained network function, which are essential for memory encoding and cognitive processing. Such preservation is particularly relevant in aging and neurodegenerative diseases where disrupted myelin and myelin axon interactions impair cognitive functions [55, 78, 79]. The absence of increased myelin density in gray matter cortical areas and in mixed white gray matter regions, such as CA1 in *Fus*^OL^cKO mice, reinforces the notion that trophic support and myelin axon communications are critical for neural function. From a translational neuroscience perspective, targeting myelin health may offer therapeutic avenues to restore or enhance cognitive functions affected by demyelinating or neurodegenerative pathology [80–82]. Future research should address how modulating myelin plasticity influences synaptic modifications and network oscillations in disease and recovery contexts.

## Supplementary Material

data1

Supplementary material available at https://doi.org/10.1088/1741-2552/ae4d8b/data1.

## Figures and Tables

**Figure 1.**
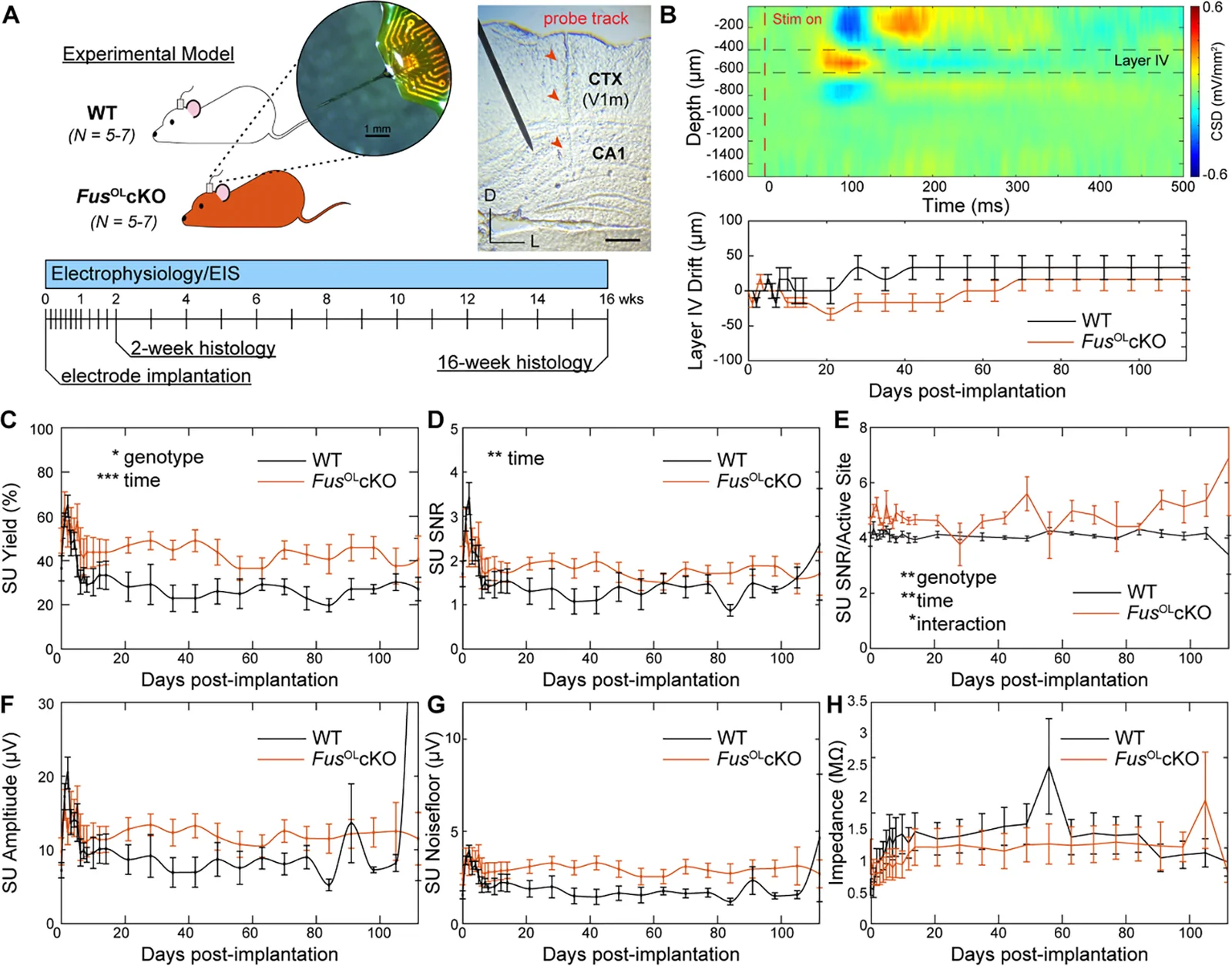
*Fus*^OL^cKO mice improve detection of individual neurons over a 16 week recording period. (A) Schematic of experimental models used and timeline of electrophysiological recording and explant histology. Inset depicts 1.6 mm long, laminar 16-channel microelectrode array. *Right*: Image of a coronal tissue section taken through a light microscope of probe track (*red arrow-heads*) extending through V1 cortex and CA1 region of the hippocampus. Scale bar = 500 *μ*m. (B) Representative current source density (CSD) plot demonstrating 500 ms of electrical current activity versus electrode implant depth following visual stimulus presentation (‘stim on’) in the visual cortex used to identify layer 4 depth (sink = red, source = blue). Average drift of layer 4 depth along implanted microelectrode shank compared to day 0 of electrode insertion between WT (*black solid*) and *Fus*^OL^cKO mice (*orange solid*). (C)–(H) Single-unit (SU) electrophysiological metrics over time between WT (*black solid*) and *Fus*^OL^cKO mice (*orange solid*). (C) Single-unit yield over time. (D) Single-unit SNR (mean peak–peak amplitude over 2*STD of noise floor). (E) SNR per electrode channel detecting a single unit (active site). (F) Single-unit amplitude over time. (G) Amplitude of noise floor over time. (H) Average impedance reported at 1 kHz over time. * indicates *p*-value < 0.05. ** indicates *p*-value < 0.01. *** indicates *p*-value < 0.001.

**Figure 2.**
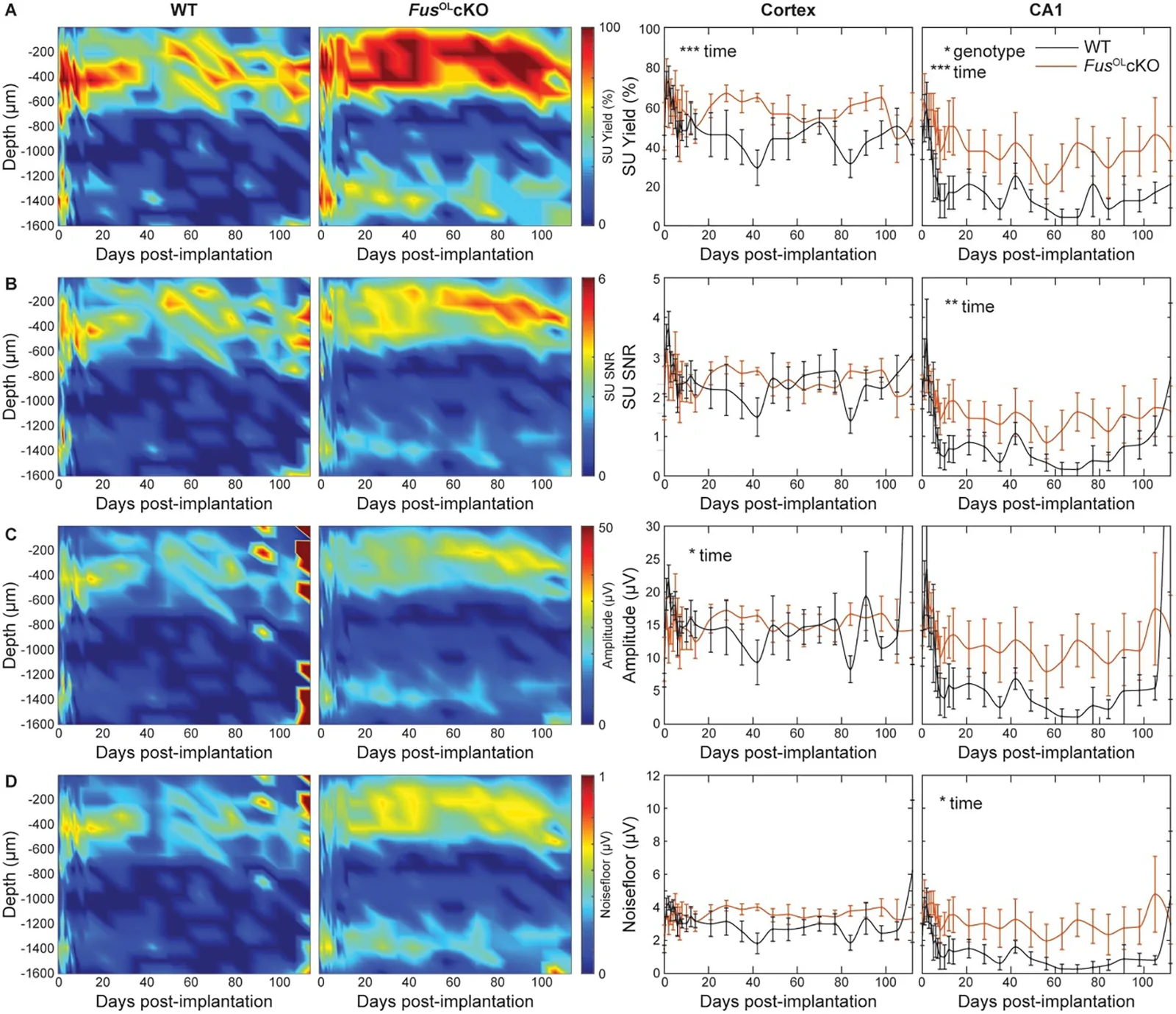
*Fus*^OL^cKO mice exhibit enhanced detection of individual neuronal waveforms within the CA1 region of the hippocampus. Single-unit (SU) metrics were plotted as averages over time and depth for *Fus*^OL^cKO mice and WT controls. Heatmaps display SU metrics as a function of time and depth averaged over all mice per genotype. Line plots demonstrate comparable differences in single-unit quality between *Fus*^OL^cKO mice and WT controls in a region-specific manner. (A) Recording yield is significantly increased in CA1 region of the hippocampus in *Fus*^OL^cKO mice compared to WT controls. (B) Signal-to-noise (SNR) of single-unit activity remains unchanged in the cortex and CA1 region of the hippocampus between *Fus*^OL^cKO mice and WT controls. (C) The amplitude of single-unit recordings remains unchanged in cortex and CA1 region of the hippocampus between *Fus*^OL^cKO mice and WT controls. (D) The noise floor of single-unit recordings remains unchanged in the cortex and CA1 region of the hippocampus between *Fus*^OL^cKO mice and WT controls. * indicates *p*-value < 0.05. ** indicates *p*-value < 0.01. *** indicates *p*-value < 0.001.

**Figure 3.**
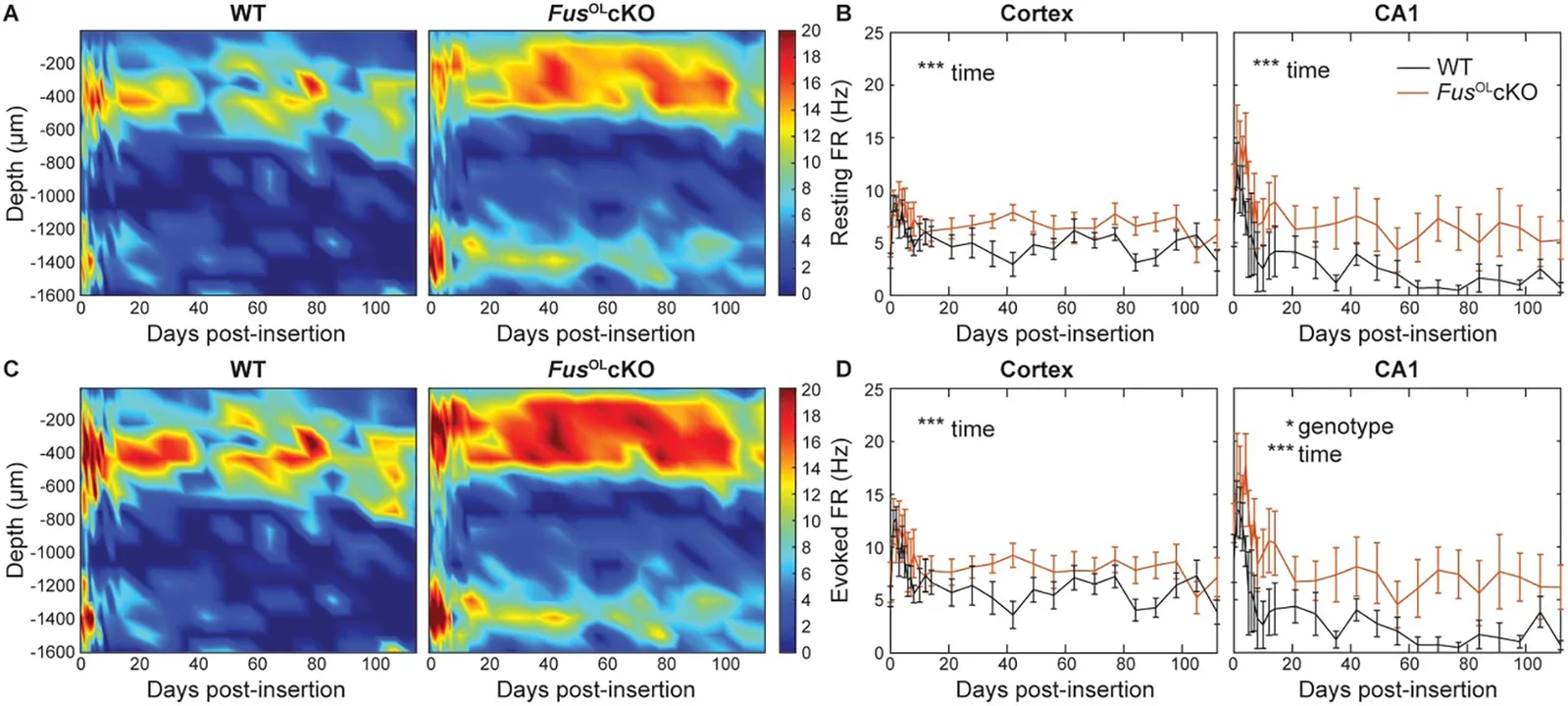
*Fus*^OL^cKO mice demonstrate elevated firing rates of individual neurons within the CA1 region of the hippocampus. Heatmaps demonstrate the average firing rate between WT and *Fus*^OL^cKO mice as a function of depth and time during (A) resting and (C) visually evoked activity. Average firing rates between WT and *Fus*^OL^cKO mice were quantified separately within cortex and subcortical CA1 region of the hippocampus for (B) resting and (D) visually evoked activity. Evoked firing rate activity significantly increased in the CA1 region, but not in the cortex, of *Fus*^OL^cKO mice compared to WT controls. * indicates *p*-value < 0.05. ** indicates *p*-value < 0.01. *** indicates *p*-value < 0.001.

**Figure 4.**
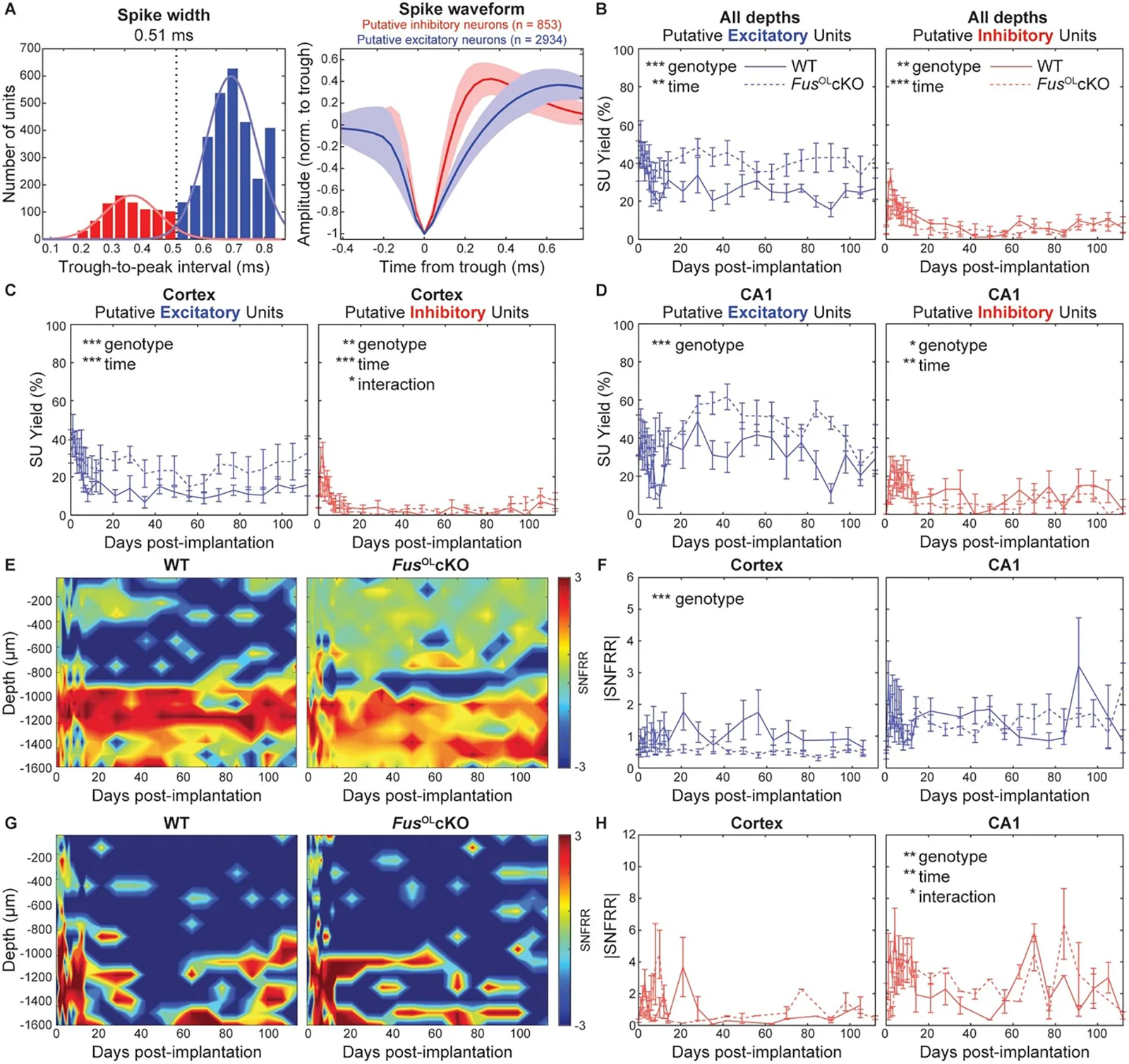
*Fus*^OL^cKO mice show selectively preserved excitatory neuron yield and responsiveness over time and across brain regions. (A) Classification of putative excitatory and inhibitory units based on trough-to-peak interval (TPI). Left: Gaussian mixture model applied to TPI distribution to determine classification threshold (0.51 ms). Right: Representative average spike waveforms (mean ± 1 SD) for excitatory (blue, TPI > 0.51 ms) and inhibitory (red, TPI < 0.51 ms) units. (B) Longitudinal analysis of excitatory and inhibitory unit yield over entire recording depth between *Fus*^OL^cKO and WT mice. (C) Longitudinal analysis of excitatory and inhibitory unit yield within the cortex between *Fus*^OL^cKO and WT mice. Excitatory yield declined over time in WT but was significantly preserved in *Fus*^OL^cKO mice. Inhibitory yield declined between both groups with limited protection conferred by hypermyelination. (D) Excitatory and inhibitory unit yield in CA1 region of hippocampus between *Fus*^OL^cKO and WT mice. Excitatory yield was preserved in *Fus*^OL^cKO mice relative to WT mice. Inhibitory yield declined over time, with WT mice showing greater yield early in the recording period. (E) Heatmap of signal-to-noise firing rate ratio (SNFRR) of excitatory units in the cortex and in CA1. (F) Average |SNFRR| of excitatory units within the cortex and CA1 region of the hippocampus in *Fus*^OL^cKO and WT mice. (G) Heatmap of signal-to-noise firing rate ratio (SNFRR) of inhibitory units in the cortex and in CA1. (H) Average |SNFRR| of inhibitory units within the cortex and CA1 region of the hippocampus in *Fus*^OL^cKO and WT mice. Inhibitory |SNFRR| in CA1 declined over time, with *Fus*^OL^cKO young mice showing transient early protection. * indicates *p*-value < 0.05. ** indicates *p*-value < 0.01. *** indicates *p*-value < 0.001.

**Figure 5.**
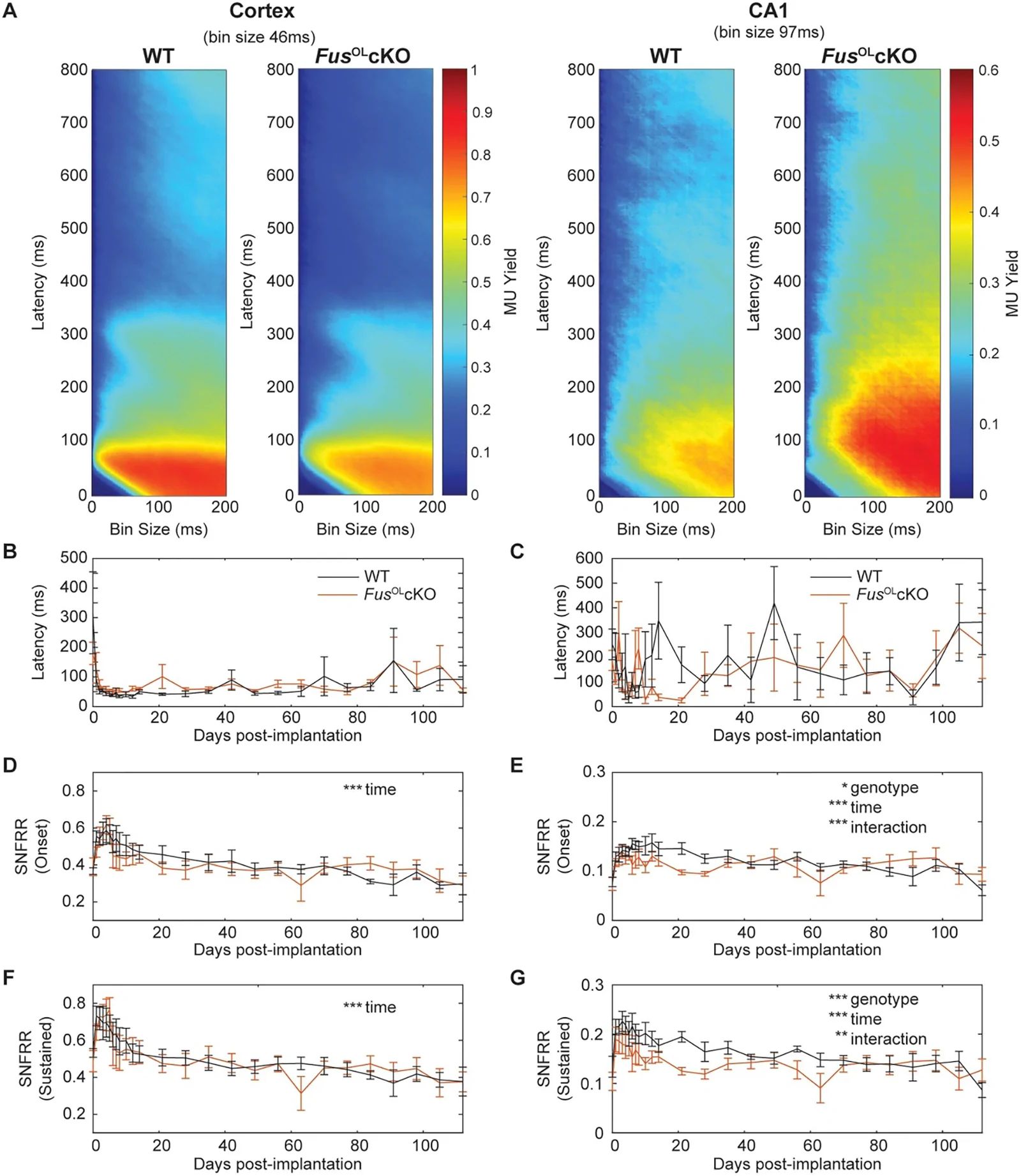
*Fus*^OL^cKO mice demonstrate reduced neuronal firing at the population-level in the CA1 region of the hippocampus. (A) Average of MU yield within the cortex and CA1 region of the hippocampus plotted in 1 ms bins as a function of latency and bin size in response to 1 s of visual stimulation for both *Fus*^OL^cKO mice and WT controls. Visual stimulation evoked a strong, transient multi-unit response within 100 ms following stimulation onset, followed by a weaker, sustained MU response between 100 and 800 ms. (B) Average latency within the cortex, calculated as the latency at which the optimal MU yield occurred in WT control mice, plotted as a function of time between *Fus*^OL^cKO mice and WT controls. (C) Average latency within the CA1 region of the hippocampus plotted as a function of time between *Fus*^OL^cKO mice and WT controls. No significant differences were observed between groups at either region. (D) Average signal-to-noise firing rate ratio (SNFRR) within the cortex during 100 ms onset response to visual stimulation between *Fus*^OL^cKO mice and WT controls. (E) Average SNFRR within the CA1 region of the hippocampus during 100 ms onset response to visual stimulation between *Fus*^OL^cKO mice and WT controls. (F) Average SNFRR within the cortex during 100–800 ms sustained response to visual stimulation between *Fus*^OL^cKO mice and WT controls. (G) Average SNFRR within the CA1 region of the hippocampus during 100–800 ms sustained response to visual stimulation between *Fus*^OL^cKO mice and WT controls. * indicates *p*-value < 0.05. ** indicates *p*-value < 0.01. *** indicates *p*-value < 0.001.

**Figure 6.**
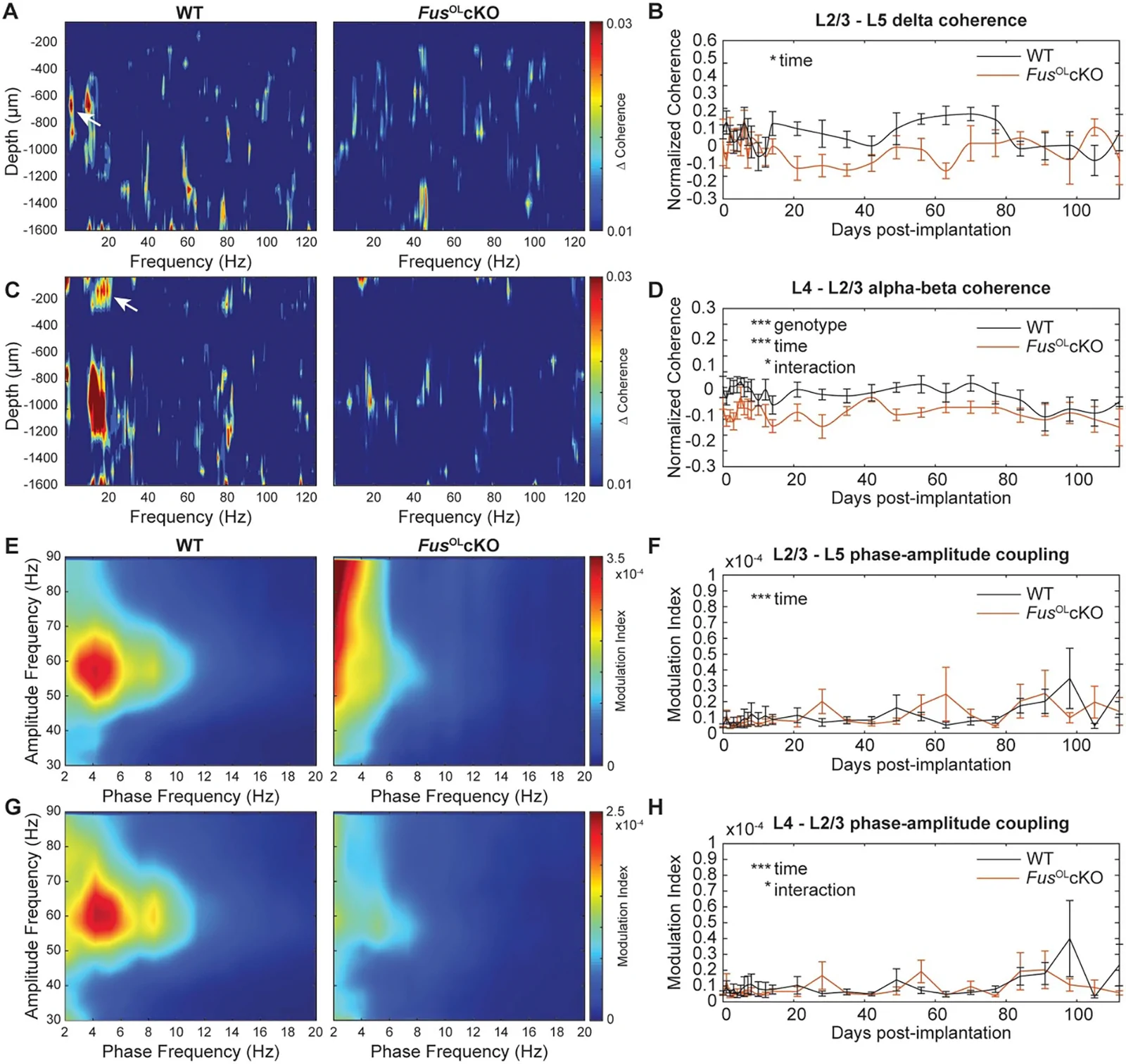
*Fus*^OL^cKO mice demonstrate reduced interlaminar connectivity within the cortex. (A) Heatmap of visually evoked change in coherence (ΔCoherence) as a function of depth and frequency demonstrated large ΔCoherence between supragranular (cortical layer 2/3) and infragranular (cortical layer 5) layers within the delta frequency range between *Fus*^OL^cKO mice and WT controls. White arrow indicates cortical layer 2/3. (B) Quantification of normalized coherence between supra- and infragranular layers within the delta frequency range over time demonstrates reduced interlaminar coherence in *Fus*^OL^cKO mice compared to WT controls. (C) Heatmap of ΔCoherence as a function of depth and frequency demonstrates large ΔCoherence between supragranular (cortical layer 2/3) and granular (layer 4) layers within the low beta/alpha-like frequency range between *Fus*^OL^cKO mice and WT controls. White arrow indicates cortical layer 4. (D) Quantification of normalized coherence between granular and supragranular layers within the low beta/alpha-like frequency range over time demonstrated reduced interlaminar coherence in *Fus*^OL^cKO mice compared to WT controls. (E) Average heatmaps of modulation index (MI) over all mice and timepoints between the phase of low-frequency oscillations and amplitude of high-frequency activity from layer 4 to layer 2/3 in *Fus*^OL^cKO and WT mice. (F) Time course of MI values between layer 4 and layer 2/3 between *Fus*^OL^cKO and WT mice. (G) Average heatmaps of MI over all mice and timepoints between layer 2/3 and layer 5 of Fus^OL^cKO and WT mice. (H) Time course of MI values between layer 2/3 and layer 5 between *Fus*^OL^cKO and WT mice. * indicates *p*-value < 0.05. *** indicates *p*-value < 0.001.

**Figure 7.**
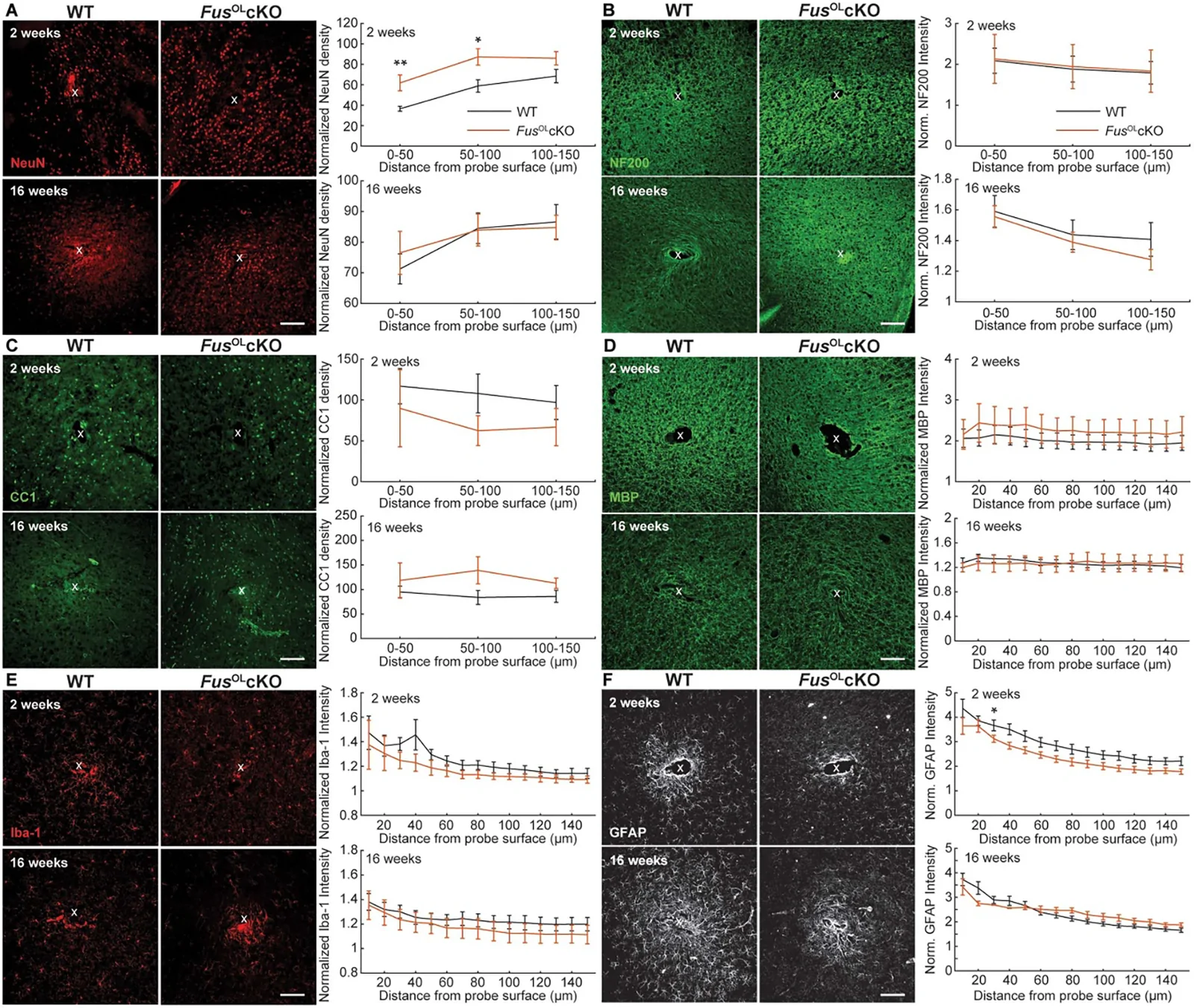
*Fus*^OL^cKO mice demonstrate early retention of neural densities in the cortex within proximity to recording electrode. (A) Representative stain of NeuN+ neurons (*red*) around a microelectrode probe hole within WT and *Fus*^OL^cKO mice at 2- and 16 weeks post-implantation demonstrated acute retention of neuronal densities within 50 *μ*m from the probe surface. (B) Representative stain of NF200+ axons (*green*) around a microelectrode probe hole within WT and *Fus*^OL^cKO mice at 2- and 16 weeks post-implantation. (C) Representative stain of CC1+ oligodendrocytes (*green*) around a microelectrode probe hole within WT and *Fus*^OL^cKO mice at 2- and 16-weeks post-implantation. (D) Representative stain of MBP+ myelin (*green*) around a microelectrode probe hole within WT and *Fus*^OL^cKO mice at 2- and 16 weeks post-implantation. (E) Representative stain of Iba-1+ microglia (*red*) around a microelectrode probe hole within WT and *Fus*^OL^cKO mice at 2- and 16 weeks post-implantation. (F) Representative stain of GFAP+ astrocytes (white) around a microelectrode probe hole within WT and *Fus*^OL^cKO mice at 2- and 16 weeks post-implantation. Scale bars = 50 *μ*m. * indicates *p*-value < 0.05. ** indicates *p*-value < 0.01.

## Data Availability

The data that support the findings of this study will be openly available following an embargo at the following URL/DOI: bioniclab.org [83].
