# Supplementary material for: Oligodendrocyte-specific fus depletion preserves CA1 single-unit fidelity and stabilizes network dynamics during chronic recording
Source: J Neural Eng. Author manuscript; Available in PMC 2026 Oct 24. (PMC13695043; doi:10.1088/1741-2552/ae4d8b)
Supplement: Supplementary file 1 [file NIHMS2214108-supplement-data1.docx]

**Supplemental Figures:** *Fus* Depleted Oligodendrocytes Improve Strength and Detection of Neuronal Activity in the CA1 Hippocampus

**Abbreviated title:** Conditional depletion of *Fus* in oligodendrocytes enhances CA1 Neuronal Recordings

**Authors:** Steven M. Wellman^1,2^, Kelly Guzman^3,4^, Naofumi Suematsu^1,2,8^, Teresa Thai^1,2^, Te-Hsuan Tung^4^, Camila Garcia Padilla^1,2^, Sadhana Sridhar^7^, Keying Chen^1,2^, Franca Cambi^3,4^, Takashi D.Y. Kozai ^1,2, 5, 6,7^*

1) Department of Bioengineering, University of Pittsburgh, Pittsburgh, PA, USA, 15213
2) Center for Neural Basis of Cognition, Pittsburgh, PA, USA, 15213
3) Veterans Administration Pittsburgh, Pittsburgh, PA, USA, 15213
4) Department of Neurology, University of Pittsburgh, Pittsburgh, PA, USA, 15213
5) Center for Neuroscience, University of Pittsburgh, Pittsburgh, PA, USA, 15213
6) McGowan Institute of Regenerative Medicine, University of Pittsburgh, Pittsburgh, PA, USA, 15213
7) Neuroscience Institute, Carnegie Mellon University, Pittsburgh, PA, USA,
8) Department of Molecular Cell Physiology, Kyoto Prefectural University of Medicine, Kyoto, Japan, 602-8566

*Corresponding author: Takashi D.Y. Kozai [tk.kozai@pitt.edu](mailto:tk.kozai@pitt.edu)

**Supplementary Figure 1. Neuron cell density and MAP2/MBP profiles in CA1 region of *Fus^OL^*cKO and WT mice**. The representative images are 20X SUMIP confocal images of stained sections collected across the CA1 subregion (2000 μm depth) of WT and *Fus*^OL^cKO mice. (A) Representative confocal images of NeuN stained CA1. Bar graphs show the NeuN+ cell density in the ipsilateral and contralateral hemisphere. (B) Representative confocal images of MAP2 stained CA1. Bar graphs show the SUM intensity in the ipsilateral and contralateral hemisphere. (C) Representative confocal images of MBP stained CA1. Bar graphs show the SUM intensity in the ipsilateral and contralateral hemisphere. Scale bars = 100 μm.

**Supplementary Figure 2. *Fus*^OL^cKO mice exhibit a greater number of excitatory synapses in CA1 subregion compared to WT controls.** *Top:* xNIS Element rendition of postsynaptic density protein 95 (PSD95, *red*) puncta, a marker of excitatory postsynaptic sites, and of vesicular glutamate transporter (vGlut1, *green*) puncta, a presynaptic excitatory neuron marker. *Bottom*: Representative confocal images of horizontal tissue sections co-stained with PSD95 and vGlut1 taken at 100X magnification to compare the number of excitatory synapses within CA1 region of hippocampus between *Fus*^OL^cKO mice and WT controls. Graph shows synapse counts/100x oil Structured Illumination Microscopy reconstructed field based on the number of pre- and post-synaptic appositions per field of view. Scale bar = 10 μm. * indicates *p*-value < 0.05.
